# Overcoming donor variability and risks associated with fecal microbiota transplants through bacteriophage-mediated treatments

**DOI:** 10.1186/s40168-024-01820-1

**Published:** 2024-07-01

**Authors:** Torben Sølbeck Rasmussen, Xiaotian Mao, Sarah Forster, Sabina Birgitte Larsen, Alexandra Von Münchow, Kaare Dyekær Tranæs, Anders Brunse, Frej Larsen, Josue Leonardo Castro Mejia, Signe Adamberg, Axel Kornerup Hansen, Kaarel Adamberg, Camilla Hartmann Friis Hansen, Dennis Sandris Nielsen

**Affiliations:** 1 Section of Food Microbiology, Gut Health, and Fermentation, Department of Food Science, University of Copenhagen, Rolighedsvej 26 4, 1958 Frederiksberg, Denmark; 2 Section of Experimental Animal Models, Department, of Veterinary and Animal Sciences, University of Copenhagen, Ridebanevej 9 1, 1871 Frederiksberg, Denmark; 3 Section of Comparative Pediatrics and Nutrition, Department of Veterinary and Animal Sciences, University of Copenhagen, Dyrlægevej 68, 1870 Frederiksberg, Denmark; 4 Department of Chemistry and Biotechnology, Tallinn University of Technology, Akadeemia tee 15, 12618 Tallinn, Estonia

## Abstract

**Background:**

Fecal microbiota transplantation (FMT) and fecal virome transplantation (FVT, sterile filtrated donor feces) have been effective in treating recurrent *Clostridioides difficile* infections, possibly through bacteriophage-mediated modulation of the gut microbiome. However, challenges like donor variability, costly screening, coupled with concerns over pathogen transfer (incl. eukaryotic viruses) with FMT or FVT hinder their wider clinical application in treating less acute diseases.

**Methods:**

To overcome these challenges, we developed methods to broaden FVT’s clinical application while maintaining efficacy and increasing safety. Specifically, we employed the following approaches: (1) chemostat-fermentation to reproduce the bacteriophage FVT donor component and remove eukaryotic viruses (FVT-ChP), (2) solvent-detergent treatment to inactivate enveloped viruses (FVT-SDT), and (3) pyronin-Y treatment to inhibit RNA virus replication (FVT-PyT). We assessed the efficacy of these processed FVTs in a *C. difficile* infection mouse model and compared them with untreated FVT (FVT-UnT), FMT, and saline.

**Results:**

FVT-SDT, FVT-UnT, and FVT-ChP reduced the incidence of mice reaching the humane endpoint (0/8, 2/7, and 3/8, respectively) compared to FMT, FVT-PyT, and saline (5/8, 7/8, and 5/7, respectively) and significantly reduced the load of colonizing *C. difficile* cells and associated toxin A/B levels. There was a potential elimination of *C. difficile* colonization, with seven out of eight mice treated with FVT-SDT testing negative with qPCR. In contrast, all other treatments exhibited the continued presence of *C. difficile*. Moreover, the results were supported by changes in the gut microbiome profiles, cecal cytokine levels, and histopathological findings. Assessment of viral engraftment following FMT/FVT treatment and host-phage correlations analysis suggested that transfer of phages likely were an important contributing factor associated with treatment efficacy.

**Conclusions:**

This proof-of-concept study shows that specific modifications of FVT hold promise in addressing challenges related to donor variability and infection risks. Two strategies lead to treatments significantly limiting *C. difficile* colonization in mice, with solvent/detergent treatment and chemostat propagation of donor phages emerging as promising approaches.

Video Abstract

**Supplementary Information:**

The online version contains supplementary material available at 10.1186/s40168-024-01820-1.

## Background

During the past decade, it has become evident that various diseases are associated with gut microbiome dysbiosis [1, 2], including recurrent *Clostridioides difficile* infections (rCDI) [3, 4]. Fecal microbiota transplantation (FMT) from a healthy donor to rCDI patients has proven highly effective in curing the disease, with a success rate exceeding 90% [5, 6]. However, FMT faces challenges such as expensive and labor-intensive donor screening [7], donor variability in terms of treatment efficacy and reproducibility [7, 8], and safety concerns since no screening methods can definitively exclude the transfer of pathogenic microorganisms from the donor. The importance of the latter was highlighted when two patients in the United States experienced severe bacterial infections after FMT, resulting in one fatality [9]. Subsequent safety alerts from the US Food & Drug Administration have warned against potential severe adverse effects associated with the transfer of pathogenic microorganisms through FMT [10, 11].

Interestingly, two independent studies [12, 13] successfully treated rCDI patients using 0.45 µm sterile filtered donor feces (containing mainly viruses but possibly also a limited fraction of intact bacteria), a method often referred to as fecal virome transplantation (FVT). The efficacy of FVT was comparable to other clinical studies using FMT (containing bacteria, etc.), suggesting that the gut virome may play an important role when treating rCDI with FMT [12, 13]. The gut virome is predominantly composed of bacteriophages (phages), which are host-specific bacterial viruses, but also include eukaryotic and archaeal viruses [14]. The concept of applying FVT from a healthy phenotype to a symptomatic phenotype has been further investigated in preclinical studies. For instance, FVT influenced the composition of the murine gut microbiome following initial perturbation with antibiotics [15]. Additionally, FVT treatment from lean donors alleviated symptoms of metabolic syndrome in three different diet-induced obesity murine models [16–18], and FVT from term piglets prevented the development of necrotizing enterocolitis in a preterm piglet model [19]. FVT has the advantage over FMT in that it significantly diminishes the transfer of viable bacteria, and FVT has recently been demonstrated to be less intrusive for both the gut microbial structure and linked to a reduced likelihood of causing harm to the jejunum in broiler chickens compared to FMT [20]. In addition to the viruses, these FVT preparations would also be expected to contain a certain level of bacterial spores and cells with sizes that allow them to pass through the 0.45 µm filter membrane pores, fecal metabolites, and extracellular vesicles of which contributions to the observed effects following FVT [12, 13, 15–21] are yet to be elucidated. Furthermore, the sterile filtration process commonly used in FVT does not eliminate the risk of transferring eukaryotic viruses, which previously have been detected in specific pathogen-free mice [22]. While it is possible to screen donor feces for known pathogenic viruses, recent studies have revealed that the human gastrointestinal tract harbors hundreds of eukaryotic viruses with unknown functions [14, 23, 24]. Most of these viruses are likely harmless to the human host, but it cannot be ruled out that they may contribute to later disease development, as exemplified by the human papillomavirus, which turned out as a significant risk factor for cervical cancer years after infection [25]. Therefore, there are good reasons to minimize the transfer of active eukaryotic viruses when applying FVT to alleviate conditions associated with gut microbiome dysbiosis, particularly when treating individuals with compromised immune systems. In contrast, phages do not actively infect and replicate in eukaryotic cells and are believed to be key players in the successful treatment of gut-related diseases using FVT/FMT [13, 16, 19, 26, 27], but the underlying mechanisms are yet poorly understood. FMT and FVT hold the potential to revolutionize treatments for many gut-related diseases, but their widespread use is unlikely due to safety concerns and donor variability. Our objective was, therefore, that we could develop different methodologies that mitigate these challenges while maintaining treatment efficacy. To generate FVTs “free” of eukaryotic viruses, we exploited the fundamental differences in characteristics between eukaryotic viruses and phages. The majority of eukaryotic viruses are enveloped RNA viruses [28, 29] and rely on eukaryotic hosts for replication. In contrast, the majority of phages are non-enveloped DNA viruses [28, 30] that require bacterial hosts for replication. A solvent/detergent method was applied to inactivate enveloped viruses (FVT-SDT), pyronin-Y was used to inhibit replication of RNA viruses (FVT-PyT), and a chemostat-propagated virome (FVT-ChP) was processed to remove the majority of eukaryotic viruses by dilution. Chemostat propagation furthermore has the advantage that it in principle allows producing more product from the same inoculum, hence increasing reproducibility. These differently processed fecal viromes were evaluated in a C57BL/6J mouse *C. difficile* infection model [31] and compared with a saline solution, FMT (previously shown to effectively treat *C. difficile* infection in a preclinical study [32]), and untreated donor-filtrated feces (FVT-UnT).

This proof-of-concept study represents an important first step towards developing safer and more consistent therapeutic approaches that can effectively target a wide range of gut-related diseases [12, 13, 16, 19, 33–35] and potentially supplement FMT with phage-mediated therapies.

## Methods

### Study design

**Fig. 1:**
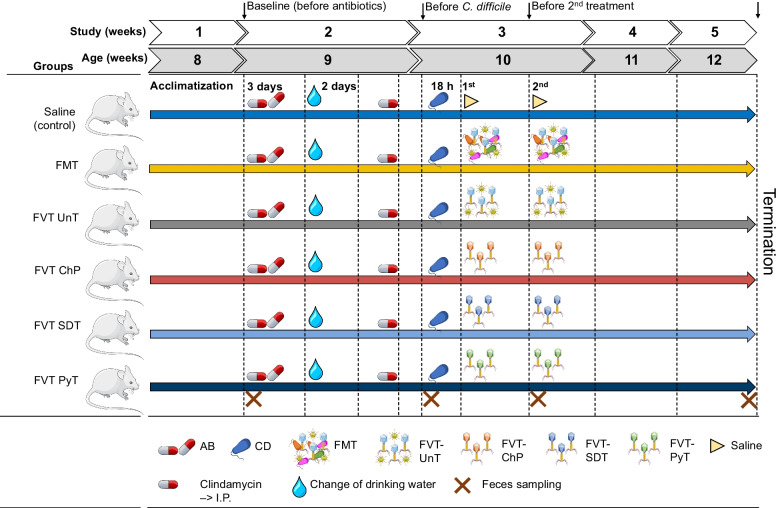
Overview of the animal model. The mice were initially treated with an antibiotic mixture (AB) in their drinking water, intraperitoneal (I.P.) injection of clindamycin, and then inoculated with *C. difficile* (CD, ~10^4^ CFU). Eighteen hours after the mice were treated with either saline (as control), FMT (fecal microbiota transplantation), FVT-UnT (fecal virome transplantation — untreated, i.e., sterile filtered donor feces), FVT-ChP (FVT chemostat propagated fecal donor virome to remove eukaryotic viruses by dilution), FVT-SDT (FVT solvent/detergent treated to inactivate enveloped viruses), and FVT-PyT (FVT pyronin-Y treated to inactivate RNA viruses). Crosses mark time points of feces sampling

The *C. difficile* infection model was based on Chen et al. [31] and accommodated the ARRIVE Essential 10 guidelines [36]. Forty-eight female C57BL/6J (JAX) mice, 8 weeks old, were obtained from Charles River Laboratories (European distributor of JAX mice) and housed at the AAALAC accredited animal facilities of the Department of Experimental Medicine (AEM, University of Copenhagen, Denmark) in Innovive disposable IVC cages that were replaced once per week. The cages were provided water, food (Altromin 1324 chow diet, Brogaarden, Denmark), bedding, cardboard housing, nesting material, felt pad, and biting stem. Upon arrival, the mice were ear tagged, randomly (simple randomization) assigned from the vendor transfer cages to the IVC cages with four mice each, and acclimatized for 1 week (Fig. 1). An antibiotic mixture (kanamycin 0.4 mg/mL, gentamicin 0.035 mg/mL, colistin 850 U/mL, metronidazole 0.215 mg/mL, and vancomycin 0.045 mg/mL) was prepared in the drinking water and provided to the mice through the IVCs for 3 days, and the water was replaced with clean antibiotic-free drinking water for 2 days. Subsequently, the mice received an intraperitoneal injection of clindamycin (2 mg/mL) diluted in sterile 0.9%(w/v) NaCl water (based on the average body weight of the mice, around 20 g). The mixture and therapeutic doses of antibiotics were performed according to the animal model described by Chen et al. [31]. The aim of the antibiotic treatment was to initiate a gut microbiome dysbiosis that increases the colonization ability of *C. difficile*. A similar sequence of events is also often observed when patients are infected with *C. difficile* during hospitalization after especially prolonged and intense antibiotic treatments [37, 38]. Twenty-four hours later, the mice were orally inoculated with 1.21 × 10^4^ CFU of *C. difficile* VPI 10463 (CCUG 19126) via oral gavage. The mice were then divided into six different treatment groups (*n* = 8): saline (positive control), FMT, FVT-UnT (untreated FVT), FVT-ChP (chemostat-propagated virome), FVT-SDT (solvent-/detergent-treated FVT for inactivating enveloped viruses), and FVT-PyT (FVT treated with pyronin-Y for inactivation of RNA viruses). The respective treatments were administered orally by gavage in two doses of 0.15 mL each (FVT solutions were normalized to 2 × 10^9^ virus-like particles (VLP)/mL), at 18 h and 72 h after *C. difficile* inoculation (Fig. 1). The sample size of eight mice per group was chosen based on previous experiments [16, 21] knowingly that the *C. difficile* infection would cause animals being euthanized at different time points, which thereby could challenge the comparability of the different timepoints and affect the statistical power. The reasoning was to accommodate the 3Rs principles (replacement, reduction, and refinement [39]) to reduce the number of animals, due to the severity level of the animal model [31], and that the chosen number of animals was sufficient to assess the potential treatment efficacy of the different processed FVTs. Also, the exclusion of a negative control group (i.e., mice not infected with *C. difficile*) was due to comparable data being available from the original study describing the model [31], again reducing the number of animals needed for the experiment. Treatments and handling of cages (cages 1–12) were performed in the order as saline, FMT, FVT-UnT, FVT-ChP, FVT-SDT, and FVT-PyT (cages 1–6) and repeated with cages 7–12 in same group order. All handling was divided between four authors (T. S. R., S. F., K. D. T., and A. V. M.) and animal caretakers to avoid confounders. All inoculations/treatments by oral gavage were performed blindedly by experienced animal caretakers at AEM. One mouse from the saline treated group (control) was euthanized immediately after oral gavage of *C. difficile*, as the culture was accidentally administered via the trachea, and one mouse from the FVT-UnT group was euthanized 1 week after the 2nd FVT treatment due to malocclusions that had led to malnutrition. This reduced these groups (saline and FVT-UnT) to *n* = 7 in both the analysis of survival probability and cytokine profile. Blinded monitoring of the animal health status was performed after *C. difficile* infection to determine if the mice had reached the humane endpoint, leading to immediate euthanization when necessary. The frequency of the health monitoring was adjusted to the current health status of the mice and ranged from every 4th hour (day and night) for the first 3 days after *C. difficile* infection, to every 8–12 h the following 4 days, and finally every 24 h for the remaining 2 weeks of recovery. The monitoring was supervised blindedly by the study veterinarian (author A. V. M.). The following qualitative parameters were used: physical activity level (i.e., decreased spontaneous or provoked activity), consistency of feces (watery or normal), body posture (hunching or normal), and whether their fur was kept clean or not. The mice were scored on a scale of 0–2: 0 (healthy), 1 (mild symptoms), and 2 (clear symptoms). Mice with a score of 2 that showed no improvement in the above parameters during the subsequent checkup were euthanized. Four authors (T. S. R., S. F., K. D. T., and A. V. M.) participated in health monitoring, euthanization, tissue, and feces sampling. Author T. S. R. was aware of the group allocations at all time points to ensure the correct treatments (but limited participation in health monitoring), while authors S. F., K. D. T., and A. V. M. were blinded at all time points. Fecal pellets were sampled whenever possible at different time points until the mice were euthanized (Fig. 1). Mouse body weights were measured at day 0, 8, 15, 16, 18, 23, 30, and 35 (Fig. S1). At the time of euthanization, samples of the intestinal content from the cecum and colon were taken. A portion of the cecum tissue was fixed in 10% neutral-buffered formalin (Sarstedt) for histological analysis and stored at room temperature. Another part of the cecum tissue, along with the intestinal content, was preserved for cytokine analysis and stored at −80 °C until use.

### *Clostridioides difficile* inoculum

*Clostridioides difficile* VPI 10463 (CCUG 19126), originating from a human infection, was used as infectious agent as in the mouse *C. difficile* infection model described by Chen et al. [31]. The bacteria were cultured in brain-heart-infusion supplement (BHIS) medium [40] with 0.02%(w/v) 1,4-dithiothreitol (Fisher Scientific) and 0.05%(w/v) L-cysteine (Fisher Scientific), grown at 37 °C in Hungate tubes (SciQuip), and handled anaerobically as previously described [41]. For solid media, 1.5%(w/v) agar (Fischer Scientific) was added. Optical density (OD_600nm_) of bacterial cultures was measured with a Genesys 30 Visible spectrophotometer (Fisher Scientific). *C. difficile* primarily synthesize its toxins during the stationary phase [42]; thus, the *C. difficile* culture in its early exponential phase was used as inoculum to minimize transfer of toxins. The *C. difficile* inoculum used for the infection was prepared as follows: a single colony of *C. difficile* was transferred to a Hungate tube containing 10 mL BHIS medium and incubated overnight at 37 °C. Then, 150 µL of the *C. difficile* overnight culture was transferred to a new Hungate tube containing 10 mL BHIS medium and incubated for 3.5 h, and its OD_600nm_ was measured. A bacterial calibration curve (OD_600nm_ versus CFU/mL) of *C. difficile* VPI 10463 was used to dilute the culture to the desired concentration (~1 × 10^5^ CFU/mL). This constituted the *C. difficile* inoculum. The exact cell concentration of the *C. difficile* inoculum (1.21 × 10^5^ CFU/mL) was evaluated with CFU counts on BHIS agar plates (Fig. S2A–D).

### Host-phage pairs

The bacteria were grown in media and at a temperature suitable for each strain (Table S1). Five phages representing different characteristics (genome, size, and structure), along with their bacterial hosts, were included to assess the influence of pyronin-Y and solvent/detergent treatment on phage activity: *Lactococcus* phage C2 (host: *Lactococcus lactic* DSM 4366), coliphage T4 (host: *Escherichia coli* DSM 613), coliphage phiX174 (host: *E. coli* DSM 13127), coliphage MS2, (host: *E. coli* DSM 5695), and *Pseudomonas* phage phi6 (host: *Pseudomonas* sp. DSM 21482). Solid media were supplemented with 1.5%(w/v) agar (Thermo Fisher Scientific) for plates and 0.7%(w/v) for soft agar. An end concentration of 10 mM MgCl_2_ and 10 mM CaCl_2_ was supplemented to media during phage propagation. Plaque activity of phages was evaluated by spot testing where 100 µL bacterial culture was mixed with 4 mL soft agar (temperature at 49 °C), poured to an agar plate, and 10 µL of a phage suspension (dilution series) was deposited on the surface of the solidified soft agar, followed by incubation according to the specific bacterial strain (Table S1).

### Epifluorescence microscopy

Virus-like particle (VLP) counts were evaluated of all fecal viromes (FVT-UnT, FVT-SDT, FVT-ChP, and FVT-PyT, Fig. S2E) by epifluorescence microscopy using SYBR Gold staining (Thermo Scientific) as described online 10.17504/protocols.io.bx6cpraw. The viral concentration was normalized using SM buffer to 2 × 10^9^ VLP/mL per treatment.

### Origin and preparation of intestinal donor material

A total of 54 male C57BL/6N mice were purchased for the purpose of harvesting intestinal content for downstream FVT/FMT applications. Upon arrival, the mice were 5 weeks old and obtained from three vendors: 18 C57BL/6NTac mice from Taconic (Denmark), 18 C57BL/6NRj mice from Janvier (France), and 18 C57BL/6NCrl mice from Charles River (Germany). We have previously experienced that a high viral diversity can be obtained by mixing the intestinal content from the same mouse strains from three different vendors, and that this approach effectively affected the gut microbiome composition of recipient mice [16, 21, 43]. The potential importance of high viral diversity on treatment outcome has also previously been suggested in relation to FMT treated *C. difficile* patients [44], and viral diversity has been reported to be positively correlated with donor phage engraftment in a human trial using FMT to treat metabolic syndrome [27]. The mice were earmarked upon arrival, randomly (simple randomization) assigned according to vendor to three cages with six mice each, and housed at the AAALAC accredited facilities at the Section of Experimental Animal Models, University of Copenhagen, Denmark, following previously described conditions [43]. They were provided with *ad libitum* access to a low-fat diet (Research Diets D12450J) for a period of 13 weeks until they reached 18 weeks of age, which was the planned termination point. Unfortunately, malocclusions resulted in malnutrition for two C57BL/6NRj mice, and they were euthanized before the intended termination date. All mice were euthanized by cervical dislocation, and samples of intestinal content (not feces pellets) from the cecum and colon were collected and suspended in 500 µL of autoclaved anoxic PBS buffer (137 mM NaCl, 2.7 mM KCl, 10 mM Na_2_HPO_4_, 1.8 mM KH_2_PO_4_). Subsequently, all samples were stored at −80 °C. In order to preserve the viability of strict anaerobic bacteria, 6 mice from each vendor (a total of 18 mice) were sacrificed and immediately transferred to an anaerobic chamber (Coy Laboratory) containing an atmosphere of approximately 93% N_2_, 2% H_2_, and 5% CO_2_, maintained at room temperature. The samples collected from these mice within the anaerobic chamber were used for FMT and anaerobic chemostat cultivation to produce the FVT-ChP. The intestinal content from the remaining 34 mice was sampled under aerobic conditions and used to generate the fecal virome for downstream processing of the FVT-UnT, FVT-SDT, and FVT-PyT treatments. A flow diagram illustrating the aforementioned processes is provided (Fig. S3). An anaerobic growth test was performed for all FVT inoculums to evaluate the level of residual viable bacterial cells or spores (Table S2). It was conducted by spreading 50 µL of undiluted FVT on non-selective Gifu Anaerobe Medium (GAM, HiMedia) 1.5%(w/v) agar plates inside an anaerobic chamber. Two replicates of each FVT were incubated at 37 °C in an anaerobic jar containing an anaerobic sachet (AnaeroGen, Thermo Fisher Scientific) outside the anaerobic chamber for 14 days before CFU counting was performed.

#### Untreated fecal virome (FVT-UnT)

For processing FVT solutions [16], thawed intestinal content from the cecum and colon was suspended in 29 mL autoclaved SM buffer (100 mM NaCl, 8 mM MgSO_4_·7H_2_O, 50 mM Tris-HCl with pH 7.5), followed by homogenization in BagPage+ 100 mL filter bags (Interscience) with a laboratory blender (Seward) at maximum speed for 120 s. The filtered and homogenized suspension was subsequently centrifuged using a centrifuge 5920R (Eppendorf) at 4500 × g for 30 min at 4 °C. The fecal supernatant was sampled for further processing of FVT solutions, while the pellet was resuspended in PBS buffer for bacterial DNA extraction. The fecal supernatant was filtered through a 0.45 µm Minisart High Flow PES Syringe Filter (Sartorius) to remove bacteria and other larger particles. This step of the FVT preparation does not definitively exclude that extracellular vesicles, bacterial cells, and spores can pass through the 0.45 µm filters. Ultrafiltration was performed to concentrate the fecal filtrate using Centriprep Ultracel YM-30K units (Millipore) that by its design constitute of an inner and outer tube. The permeate in the inner tube was discarded several times during centrifugation at 1500 × g at 20 °C until approximately 0.5 mL was left in the outer tube, which at this point was considered as a fecal virome. The 30 kDa filter from the Centriprep Ultracel YM-30K units was removed with a sterile scalpel and added to the fecal virome to allow viral particles to diffuse overnight at 4 °C. In order to trace back the origin of specific bacterial or viral taxa, the fecal viromes were mixed based on cages, taking into account the coprophagic behavior of mice [45]. The ultrafiltration of FVT-UnT, FVT-ChP, FVT-SDT, and FVT-PyT is expected to remove the vast majority of metabolites below 30 kDa [46, 47]. These fecal viromes were mixed into one final mixture from mice of all three vendors representing the “untreated fecal virome,” FVT-UnT, which was immediately stored at −80 °C. The remaining fecal viromes were stored at 4 °C prior to downstream processing to inactivate the eukaryotic viruses in the fecal viromes by either dissolving the lipid membrane of enveloped viruses with solvent/detergent treatment or inhibit replication of RNA viruses with pyronin-Y.

#### Solvent/detergent treated fecal virome (FVT-SDT)

The solvent/detergent treatment is commonly used to inactivate enveloped viruses, as most eukaryotic viruses possess an envelope, while non-enveloped viruses, including phages, are not affected by this treatment [48, 49]. Following the guidelines set by the World Health Organization (WHO) [50] and Horowitz et al. [48] for the clinical use of solvent/detergent treated plasma, the fecal viromes were subjected to incubation in a solution containing 1%(w/v) tri(n-butyl) phosphate (TnBP) and 1%(w/v) Triton X-100 at 30 °C for 4 h. It is important to note that the majority of inactivation typically occurs within the first 30–60 min of the solvent/detergent treatment [50]. The removal of TnBP and Triton X-100 was performed according to the method described by Treščec et al. [51]. In brief, the applied volume of Amberlite XAD-7 in the column was set to 150% of the theoretical binding capacity to ensure a sufficient removal of TnBP and Triton X-100. The resin column was equilibrated with 0.01 M phosphate buffer (Na_2_HPO_4_ and NaH_2_PO_4_) pH 7.1 containing 0.5 M NaCl until OD_280nm_ was < 0.02. Each solvent/detergent treated fecal virome (mixed by cage) was added separately to the column, and OD_280nm_ was measured to follow the concentration of proteins (expected viral particles and other metabolites > 30 kDa) and until OD_280nm_ was < 0.02. A 0.01 M phosphate buffer containing 1 M NaCl was used to release potential residual particles from the resin [51]. The removal of the solvent/detergent agents from the fecal viromes yielded approx. 100 mL viral flow-through from the column which was concentrated to 0.5 mL using Centriprep Ultracel YM-30K units as described in the previous section. The final product constituted the FVT-SDT treatment and was stored at −80 °C until use.

#### Pyronin-Y treated fecal virome (FVT-PYT)

Pyronin-Y (Merck) is a strong red-colored fluorescent compound. It has been reported to exhibit efficient binding to single-stranded and double-stranded RNA (ss/dsRNA), while its binding to single-stranded and double-stranded DNA (ss/dsDNA) is less effective [52, 53]. Initial screening was conducted to determine the optimal conditions for viral inactivation using various concentrations of pyronin-Y, different incubation times, and temperatures for RNA and DNA phages. The fecal filtrate was treated with 100 µM pyronin-Y and incubated at 40 °C overnight to inactivate viral particles containing RNA genomes. To remove the pyronin-Y molecules that were not bound to particles, the pyronin-Y treated fecal filtrate suspensions were diluted in 50 mL SM buffer and subsequently concentrated to 0.5 mL using ultrafiltration with Centriprep Ultracel YM-30K units. This process was repeated three times, resulting in a transparent appearance of the pyronin-Y treated fecal filtrate, which constituted the FVT-PyT treatment and was stored at −80 °C until use.

#### Fecal microbiota transplantation (FMT)

The mouse intestinal content that was sampled anoxically (Fig. S3) was diluted 1:20 in an anoxic cryoprotectant consisting of PBS buffer and 20% (v/v) glycerol and stored at −80 °C until use.

#### Chemostat-propagated fecal virome (FVT-ChP)

The preparation of the chemostat-propagated virome was performed as described previously [54]. Briefly, anaerobic handled mouse cecum content was utilized for chemostat propagation of the donor phageome. The culture medium was formulated to resemble the low-fat diet (Research Diets D12450J) provided to the donor mice as their feed (Table S3), and growth conditions such as temperature (37 °C) and pH (6.4) were set to simulate the environmental conditions present in the mouse cecum. The end cultures, which underwent fermentation with a slow dilution rate (0.05 volumes per hour), exhibited a microbial composition that resembled the initial microbial composition profile of the donor [54]. These batches were combined to form the FVT-ChP treatment and were stored at −80 °C until use.

### Cytokine analysis

Pre-weighted cecum tissue was homogenized in 400 μL lysis buffer (stock solution: 10 mL Tris lysis buffer, 100 μL phosphatase inhibitor 1, 100 μL phosphatase inhibitor 2, and 200 μL protease inhibitor) (MSD inhibitor pack, Meso Scale Discovery) using a FastPrep Bead Beater Homogenizer (MP Biomedicals) and centrifuged (8000 × g; 4 °C; 5 min). Samples were diluted 1:2 and analyzed for IFN-γ, GM-CSF, IL-15, IL-6, IL-10, KC/GRO, MIP-2, TNF-α IL-17A/F, and IL-22 in a customized metabolic group 1 U-PLEX (MSD) according to manufacturer’s instructions. Samples were analyzed using the MESO QuickPlex SQ 120 instrument (Meso Scale Discovery), and concentrations were extrapolated from a standard curve using Discovery Workbench v.4.0 (Meso Scale Discovery) software. Measurements out of detection range were assigned the value of lower (set to 0) or upper detection limit. The cytokine analysis was performed by a blinded investigator (author C.H.F.H.).

### Histology and cytotoxicity assay

Formalin-fixed, paraffin-embedded cecum tissue sections were stained with hematoxylin and eosin for histopathological evaluation by a blinded investigator (author A. B.). A composite score was assigned, taking into account the following pathological features: (1) immune cell infiltration, (2) submucosal edema or hemorrhage, and (3) epithelial injury, each with a range of severity/extent as follows: 0: none, 1: mild, 2: moderate, 3: severe) for a cumulative pathology grade between 0 and 9 [31]. Cecum tissue samples with mechanical damage were excluded for the analysis.

The RIDASCREEN *C. difficile* Toxin A/B ELISA kit (R-Biopharm) was used to measure the toxin concentrations in the mice feces by following the instructions of the manufacturer. The OD_450nm_ was measured with a Varioskan Flash plate reader (Thermo Fisher Scientific).

### qPCR measuring *C. difficile* abundance

*C. difficile* in the fecal samples was enumerated using quantitative real-time polymerase chain reaction (qPCR) with species-specific primers (C.Diff ToxA Fwd: 5′-TCT ACC ACT GAA GCA TTA C-3′, C.Diff ToxA Rev: 5′-TAG GTA CTG TAG GTT TAT TG-3′ [55]) purchased from Integrated DNA Technologies. Standard curves were based on a dilution series of total DNA extracted from a monoculture of *C. difficile* VPI 10463. The qPCR results were obtained using the CFX96 Touch Real-Time PCR Detections System (Bio-Rad Laboratories) and the reagent RealQ plus 2× Master Mix Green low ROX (Amplicon) as previously described [56].

### Preprocessing of fecal samples for separation of viruses and bacteria

Fecal samples from three different time points were included to investigate gut microbiome changes over time: baseline (before antibiotic treatment), before *C. difficile* infection (after antibiotic treatment), and at termination or at euthanization. This represented in total 142 fecal samples. Separation of the viruses and bacteria from the fecal samples generated a fecal pellet and fecal supernatant by centrifugation and 0.45 µm filtering as described previously [43], except the volume of fecal homogenate was adjusted to 5 mL using SM buffer.

### Bacterial DNA extraction, sequencing, and data preprocessing

The DNeasy PowerSoil Pro Kit (Qiagen) was used to extract bacterial DNA from the fecal pellet by following the instructions of the manufacturer. The final purified DNA was stored at −80 °C, and the DNA concentration was determined using Qubit HS Assay Kit (Invitrogen) on the Qubit 4 Fluorometric Quantification device (Invitrogen). The bacterial community composition was determined by Illumina NextSeq based high-throughput sequencing of the 16S rRNA gene V3 region, as previously described [43]. Quality control of reads, de-replicating, purging from chimeric reads, and constructing zero-radius operational taxonomic units (zOTU) were conducted with the UNOISE pipeline [57] and taxonomy assigned with Sintax [58] using the EzTaxon for 16S rRNA gene database [59]. zOTU represents unique sequence variants where only sequence alignments with 100% similarity are merged into the same zOTU. Code describing this pipeline can be accessed in https://github.com/jcame/Fastq_2_zOTUtable. The average sequencing depth after quality control (accession: PRJEB58777, available at ENA) for the fecal 16S rRNA gene amplicons was 60,719 reads (min. 11,961 reads and max. 198,197 reads).

### Viral RNA/DNA extraction, sequencing, and data preprocessing

The sterile-filtered fecal supernatant was concentrated using Centrisart centrifugal filters with a filter cutoff at 100 kDA (Sartorius) by centrifugation at 1500 × g at 4 °C (10.17504/protocols.io.b2qaqdse). The fecal supernatant (140 µL) was treated with five units of Pierce Universal Nuclease (Thermo Fisher Scientific) for 10 min at room temperature prior to viral DNA extraction to remove free DNA/RNA molecules. The viral DNA/RNA was extracted from the fecal supernatants using the Viral RNA Mini Kit (Qiagen) as previously described [43, 60]. Reverse transcription was executed with SuperScript IV VILO Master Mix by following the instructions of the manufacturer and subsequently cleaned with DNeasy blood and tissue kit (Qiagen) by only following steps 3–8 in the instructions from the manufacturer. In brief, the DNA/cDNA samples were mixed with ethanol, bound to the silica filter, washed two times, and eluted with 40 µL elution buffer. Multiple displacement amplification (MDA, to include ssDNA viruses) using GenomiPhi V3 DNA Amplification Kit (Cytiva) and sequencing library preparation using the Nextera XT kit (Illumina) were performed at previously described [43] and sequenced using the Illumina NovaSeq platform at the sequencing facilities of Novogene (Cambridge, UK). The average sequencing depth of raw reads (accession: PRJEB58777, available at ENA) for the fecal viral metagenome was 17,384,372 reads (min. 53,960 reads and max. 81,642,750 reads). Using Trimmomatic v0.35, raw reads were trimmed for adaptors, and low-quality sequences (< 95% quality, < 50 nt) were removed. High-quality reads were de-replicated and checked for the presence of PhiX control using BBMap (bbduk.sh) (https://www.osti.gov/servlets/purl/1241166). Virus-like particle-derived DNA sequences were subjected to within-sample *de novo* assembly only using Spades v3.13.1, and contigs with a minimum length of 2200 nt, were retained. Contigs from all samples were pooled and dereplicated by chimera-free species-level clustering at ~95% identity using the script described in [61] and available at https://github.com/shiraz-shah/VFCs. Contigs were classified as viral by VirSorter2 [62] (“full” categories | dsDNAphage, ssDNA, RNA, Lavidaviridae, NCLDV | viral quality = 1), VIBRANT [63] (high-quality | medium-quality | complete), CheckV [64] (high-quality | medium-quality | complete), and VirBot [65]. Any contigs not classified as viral by any of the four software’s were discarded. The taxonomical categories of “Other,” “Unclassified virus,” and “Unknown” that are used in the different figures are different entities. “Other” encompasses all remaining low abundance taxa not depicted in the plot. “Unknown” refers to contigs that may be viruses but lack specific data records confirming their viral origin, and “Unclassified virus” represents viruses that have been identified as having viral origin but could not be further classified. Taxonomy was inferred by blasting viral ORFs against a database of viral proteins created from the following: VOGDB v.217 (vogdb.org), NCBI (downloaded 14/10/2023), COPSAC [61], and an RNA phage database [66], selecting the best hits with a minimum *e*-value of 10e^−6^. Phage-host predictions were done with IPhoP [67], which utilizes a combination of different host predictors. Following assembly, quality control, and annotations, reads from all samples were mapped against the viral (high-quality) contigs (vOTUs) using bowtie2 [68] and a contingency table of contig length and sequencing depth normalized reads, here defined as vOTU table (viral contigs). Code describing this pipeline can be accessed in https://github.com/frejlarsen/vapline3. Mock phage communities (phage C2, T4, phiX174, MS2, and phi6, Table S1) were used to both spike the FVT inoculums and as positive controls (normalized to ~10^6^ PFU/mL for each phage) for virome sequencing to validate the sequencing protocol’s ability to include the different genome types of ssDNA, dsDNA, ssRNA, and dsRNA.

### Bioinformatics of bacterial and viral sequences and statistical analysis

The dataset was first cleaned to remove zOTUs/viral contigs found in less than 5% of the samples. Despite this, the resulting dataset retained over 99.8% of the total reads. R version 4.3.2 was used for subsequent analysis and presentation of data. A minimum threshold of sequencing reads for the bacteriome and virome analysis was set to 2200 reads and 15,000 reads, respectively. The main packages used were phyloseq [69], vegan [70], DESeq2 [71], ampvis2 [72], ggpubr, psych, igraph, ggraph, pheatmap, ComplexHeatmap, and ggplot2. Potential contaminations of viral contigs were removed by read count detected in negative controls through R package microDecon [73] (runs = 1, regressions = 1), and 35.1% of entries were removed. Cumulative sum scaling (CSS) was applied for the analysis of *β*-diversity. CSS normalization was performed using the metagenomeSeq package. *α*-diversity analysis (Shannon diversity index) was based on raw read counts for bacteriome analysis, while the virome read counts were normalized on the basis of transcripts per million (TPM), and statistics were based on ANOVA. *β*-diversity was represented by Bray-Curtis dissimilarity, and statistics were based on PERMANOVA. DESeq2 was used to identify differential abundant taxa on the summarized bacterial species level and viral contigs (vOTUs) level. The correlation heatmap between bacterial zOTUs and vOTUs (viral contigs) were calculated using pairwise Spearman’s correlations and FDR corrected. Cytokine levels, toxin levels, *C. difficile* abundance, and histology data were analyzed in R using linear models with saline as control group, while the log-rank test was used to compare the survival distributions and FDR was used for corrections with multiple testing. Comparisons of means where used to calculate differences in PFU counts (https://www.medcalc.org/calc/comparison_of_means.php).

## Results

We here hypothesized that different methodologies could be applied to overcome the challenges of donor variability and infection risks of eukaryotic viruses that are associated with FVT/FMT while maintaining the treatment efficacy that previously has been reported for FVT/FMT treated recurrent *C. difficile* infection (rCDI) patients [5, 12, 13]. To produce “eukaryotic virus-free” fecal viromes, we developed methodologies that utilized fundamental differences in characteristics between eukaryotic viruses and phages: the majority of eukaryotic viruses are enveloped RNA viruses [28, 29] and require eukaryotic hosts for replication, while the majority of phages are non-enveloped DNA viruses [28, 30] and require bacterial hosts for replication. A solvent/detergent method was applied to inactivate enveloped viruses (FVT-SDT), pyronin-Y was used to inhibit replication of RNA viruses (FVT-PyT), and a chemostat-propagated virome (FVT-ChP) was created to remove the majority of eukaryotic viruses by dilution [54]. These differently processed fecal viromes were as a proof of concept tested in a murine *C. difficile* infection model (Fig. 1) and compared with a saline solution (sham treatment), FMT, and untreated FVT (FVT-UnT). All treatments originated from the same intestinal donor content (and not from fecal pellets).

### Evaluation of methodologies applicability to inactivate enveloped and RNA viruses

**Fig. 2:**
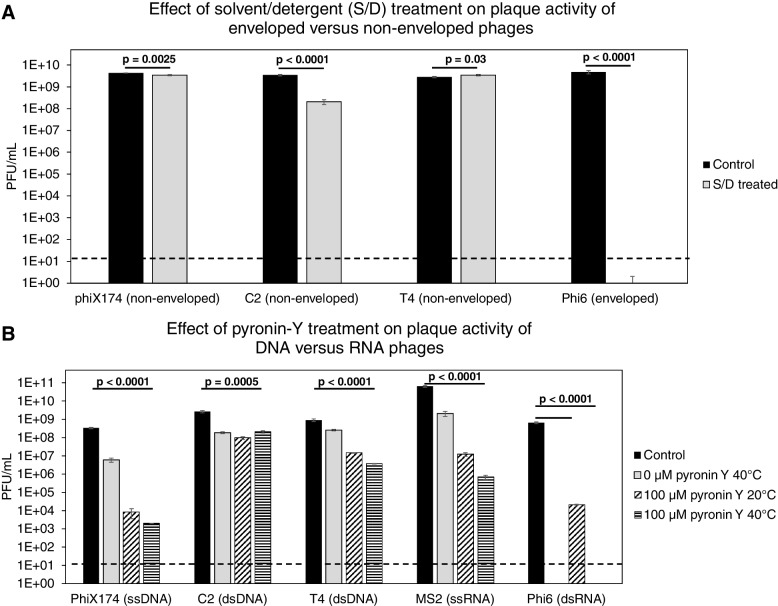
Evaluation of inactivation of phage activity (plaque-forming units per mL, PFU/mL) with solvent/detergent or pyronin-Y treatment was evaluated on their respective bacterial hosts all performed in three replicates. Significance was evaluated with comparisons of means and *t*-test, with only considering the comparison between the control and the treatment applied in the study. **A** Three non-enveloped phages (phiX174, C2, and T4) and one enveloped phage (phi6) were treated with solvent/detergent (S/D), and their plaque activity was evaluated on their respective bacterial hosts. **B** Phages representing ssDNA (phiX174), dsDNA (C2 and T4), ssRNA (MS2), and dsRNA (phi6) were treated with pyronin-Y, and their plaque activity at different incubation conditions was evaluated on their respective bacterial hosts. Dashed lines mark the detection limit of the applied assay

The applicability of the solvent/detergent and pyronin-Y treatments to inactivate eukaryotic viruses while maintaining phage activity was tested by using phages representing different characteristics, such as enveloped (phi6) versus non-enveloped (phiX174, T4, and C2) phage structure and ss/dsDNA (phiX174, T4, C2) versus ss/dsRNA (MS2, phi6) genomes (Fig. 2A & B). The solvent/detergent treatment completely inactivated phage activity (as determined by plaque-forming units (PFU)/mL) of the enveloped phage phi6 from 10^9^ PFU/mL to below the detection limit (*p* < 0.0001). The activity of the non-enveloped phages phiX174 and T4 was largely unaffected with less than 0.1 log_10_ decrease, whereas phage C2 showed a 1 log_10_ decrease in PFU/mL (*p* < 0.0001, Fig. 2A). Pyronin-Y was used to inactivate the replication of viruses harboring RNA genomes. Based on numerous combinations of pyronin-Y concentrations, temperatures, and incubation time, an overnight incubation at 40 °C with 100 µM pyronin-Y was chosen. This treatment reduced the ssRNA phage MS2 with 5 log_10_ PFU/mL (*p* < 0.0001) and dsRNA phage phi6 with more than 4 log_10_ PFU/mL (*p* < 0.0001) at 20 °C. Phi6 showed to be temperature sensitive since incubation at 40 °C alone inactivated (*p* < 0.0001) this enveloped phage. The plaque-forming ability of phages C2 (dsDNA), T4 (dsDNA), and phiX174 (ssDNA) was unfortunately also affected by the pyronin-Y treatment at 40 °C, with a decrease of 1, 2.5, and 5 log_10_ PFU/mL (*p* < 0.0005, Fig. 2B), respectively. Thus, it would be expected that a notable fraction of the phages in the FVT will be inactivated using the pyronin-Y treatment. In a parallel study, we showed that the chemostat propagation of fecal viromes minimized the relative abundance of eukaryotic viruses [54].

### Fecal viromes maintained high treatment efficacy after inactivation of enveloped viruses

**Fig. 3:**
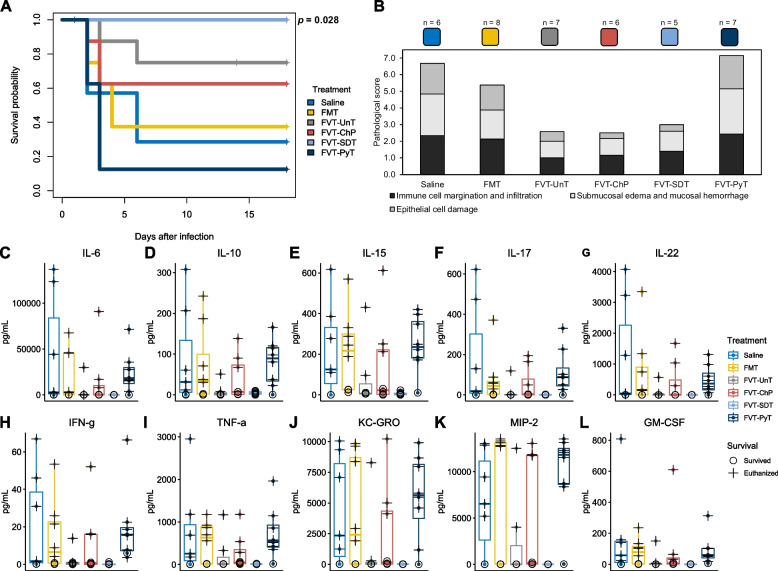
Overview of mouse phenotypic characteristics. **A** Kaplan-Meier curve showing the survival probability of the mice that was associated with the different treatments when compared with the saline treated group. Pairwise comparisons between treatment groups with corrections for multiple testing were performed. **B** Pathological score of cecum tissue evaluating the effect of the treatments’ ability to prevent *C. difficile* associated damage of the cecum tissue. **C** to **L** Showing the overall cytokine profile in the mouse cecum tissue of the different treatments. The euthanized (cross) mice are differentiated from the mice that survived (circle) the *C. difficile* infection and therefore represent two different time points. It was not possible to perform statistical analysis for the pathological score and cytokine profiles due to different timepoints of sampling of the euthanized and survived animals

As a main endpoint parameter, the treatment efficacy of the FMT/FVTs, specifically preventing the mice from reaching the humane endpoint, was assessed in a murine *C. difficile* infection model (Fig. 1). The survival probability rate associated with the different treatments was evaluated using a Kaplan-Meier estimate (Fig. 3A) and compared to the mice treated with saline (2/7 mice). The analysis revealed a significantly improved survival rate (8/8 mice, *p* = 0.03) for mice treated with FVT-SDT, while the FVT-UnT (5/7 mice)- and FVT-ChP (5/8 mice) treated mice showed tendencies (*p* < 0.24) of numerical, but non-significant, improvements of their survival rate. On the other hand, the FVT-PyT-treated mice and, unexpectedly, the FMT treatment showed no improvement in survival rate (1/8 and 3/8 mice, respectively).

The pathological score (Fig. 3B & Fig. S4A–J) and the levels of 10 pro- and anti-inflammatory cytokines of the cecum tissue were evaluated of both the mice that reached the humane endpoint and the mice that survived until study termination, which made it difficult to statistically evaluate these measures of the different sampling time points. The cecal histopathology and cytokine profiles therefore generally reflected whether the mice survived (with low or no inflammatory response when measured at study termination, marked with a circle) or were euthanized (with a high inflammatory response, marked with a cross) due to *C. difficile* infection (Fig. 3C–L). Thus, these measures were included to evaluate the animal’s disease status/recovery at termination or euthanization as well as the different FVT/FMT treatment abilities to increase the chances for the animals of not reaching the humane endpoint. However, a decrease in the average pathological score and cytokine levels supported the qualitative health evaluations (Fig. S4B–G) and the treatment efficacy associated with the improved survival rate of FVT-UnT, FVT-SDT, and FVT-ChP treated mice compared to the saline control, FMT, and FVT-PyT (Fig. 3B & Fig. S4A). The average pathological score at 6.7 of the saline treatment was in line with the original published *C. difficile* infection mouse model that reported a pathological score at 7.0 for *C. difficile*-infected mice, compared to mice not infected with *C. difficile* that showed a score at 1.3 [31]. Overall, the FVT-SDT appeared as the superior treatment to prevent severe infections of *C. difficile*, since all eight out of eight mice did not reach the humane endpoint.

### Successful treatments impede *C. difficile* colonization and subsequent disease development

**Fig. 4:**
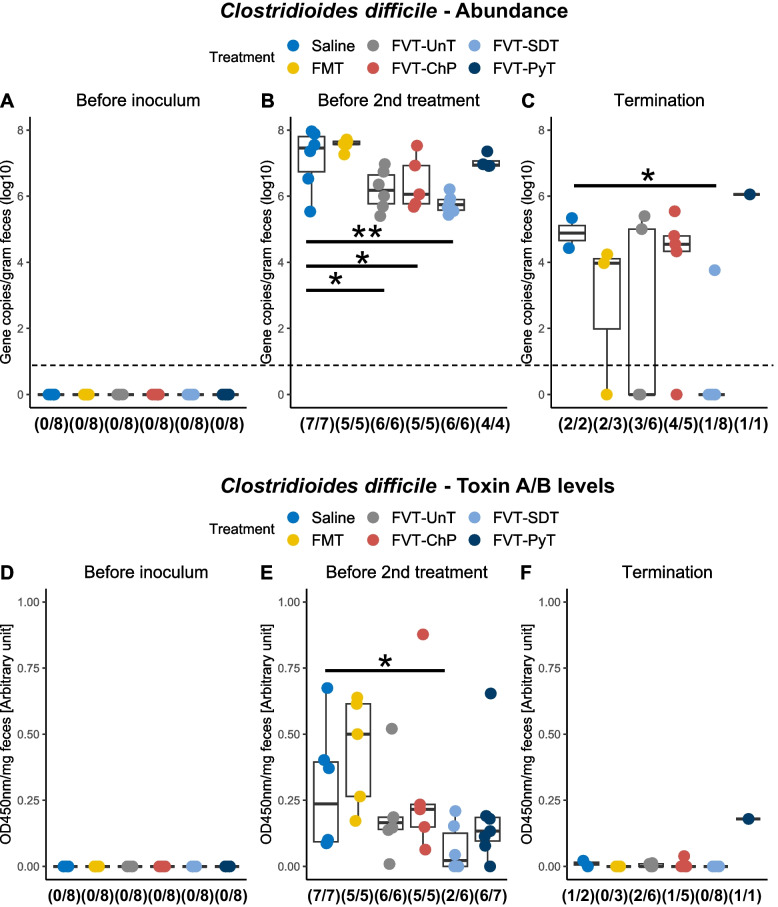
Evaluation of *C. difficile* abundance **A** to **C** by qPCR targeting the *toxA* gene and **D** to **F** the associated toxin A/B levels measured with ELISA on feces samples from three different time points: before *C. difficile* inoculum, before 2nd treatment, and at study termination. The fraction below the boxplots highlights the number of mice that was detected positive of either *C. difficile* or toxin A/B. Dashed line marks the detection limit of the applied assay. *p < 0.05, **p < 0.01

*C. difficile* abundance in feces was quantified using qPCR (Fig. 4A–C) to evaluate the infectious load at different time points. No *C. difficile* was detected before inoculation with *C. difficile* (Fig. 4A). The FVT-SDT treated mice exhibited an average of 2 log_10_ lower *C. difficile* abundance (*p* = 0.001) (gene copies per gram feces) compared to the saline-treated mice before the 2nd treatment, and the FVT-UnT (*p* = 0.013) and FVT-ChP (*p* = 0.039) treatments resulted in a 1.5 log_10_ lower abundance. This suggested that these three treatments effectively impeded *C. difficile* colonization in the gut. In contrast, the FMT- and FVT-PyT-treated mice had similar *C. difficile* abundance as the saline treated group. A possible clearance of *C. difficile* colonization was observed at study termination, as seven out of eight FVT-SDT treated mice tested negative for *C. difficile*, while all other treatments showed persistency of *C. difficile* (Fig. 4C). The levels of the *C. difficile*-associated toxin A/B were measured using an ELISA based assay, which showed similar patterns as the qPCR data (Fig. 4D–F). Just before the 2nd treatment, the toxin A/B levels in the FVT-SDT treated mice were significantly lower than those in the saline group (*p* < 0.05), and only two mice in the FVT-SDT group exhibited detectable toxin A/B. In contrast, toxin A/B was detected in all mice in the other FMT/FVT treatments and control (Fig. 4E). At termination, toxin A/B could not be detected in any of the FVT-SDT treated mice but was detected in a fraction of mice in the other treatment groups (Fig. 4F). The decrease in the abundance of *C. difficile*, the causing pathogenic agent (Fig. 4A–C), along with the diminished levels of toxin A/B (Fig. 4D–F) observed in the FVT-UnT, FVT-SDT, and FVT-ChP groups in comparison to mice treated with saline, aligns with the corresponding higher survival rates (Fig. 3A). This suggests a supportive relationship between reduced pathogen presence and toxin levels with improved overall chances of survival.

### Fecal virome treated with solvent/detergent supports recovery of the bacterial community in a dysbiotic gut microbiome

**Fig. 5:**
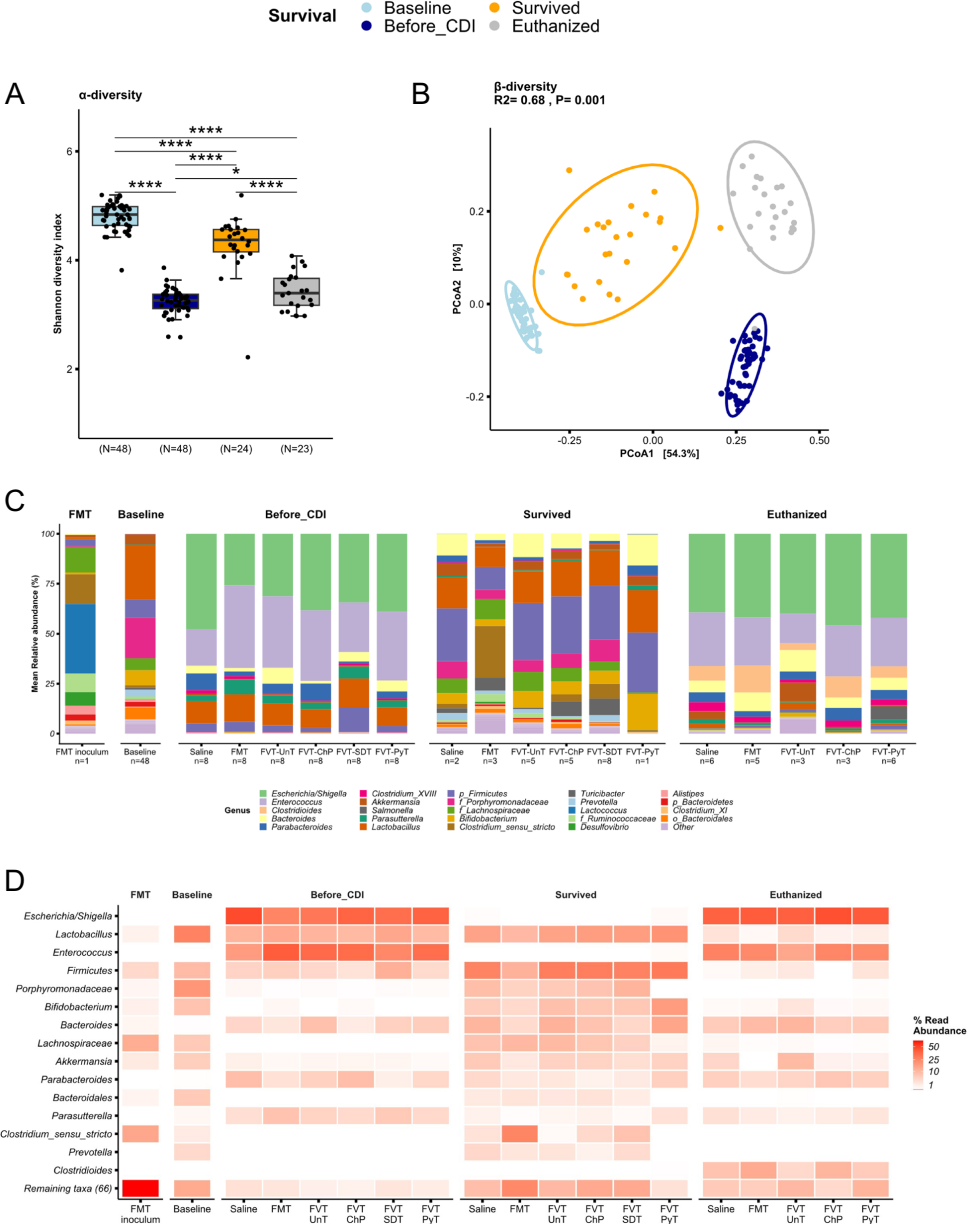
Bacteriome analysis based on 16S rRNA gene amplicon sequencing. **A** The bacterial Shannon diversity index (α-diversity) and **B** Bray-Curtis dissimilarity based PCoA plot (β-diversity) at baseline (before antibiotic treatment), before *C. difficile* infection (after antibiotic treatment), for the mice that survived *C. difficile* infection (regardless of the treatment), and the euthanized mice. **C** Bar plot and **D** heatmap illustrating the bacterial relative abundance in percentage of the dominating bacterial taxa that was associated with the different mice that either survived the infection or were euthanized. The “n” below the bar plots highlights the number of mice of which the taxonomical average was based on. *p < 0.05, ****p < 0.0005

The gut microbiome analysis included three time points: baseline (before antibiotics), before *C. difficile* infection (after antibiotic treatment), and at planned termination or if the mice reached the humane endpoint prior study termination. The latter posed the inherent comparability challenge by the time difference between the euthanized mice and the mice that survived the infection (Fig. S5). Thus, these gut microbiome profiles reflected whether the mice survived the infection or were euthanized. Despite this, we deliberately included the gut microbiome data for all three time points to facilitate a comparative analysis for answering the following two main questions: (1) Did the different FVT/FMT treatments contribute to the restoration of the gut microbiome relative to baseline? (2) Which significant changes characterized the gut microbiome at the time of the euthanized and survived mice compared with the time point before the *C. difficile* infection? To do so, we verified that there were no initial differences (*p > 0.3*) in the bacterial and viral gut microbiome profiles at both baseline (before antibiotics) and before *C. difficile* infection (after antibiotic treatment) between the mice that were later either euthanized or survived the *C. difficile* infection (Fig. 5 & Fig. S6). The antibiotic intake through the drinking water was similar across the cages (Table S4). The overall bacterial composition (Bray-Curtis dissimilarity) and diversity (Shannon diversity index) were significantly different (*p* < 0.05) between the different time points (Fig. 5A–B); however, the mice that survived the infection tended to be more similar to baseline, compared with the time before *C. difficile* infection and the euthanized mice. The bacterial taxonomic profile of the mice that had survived the infection was dominated by lactobacilli, *Prevotella*, *Clostridium sensu stricto*, *Bacteroides*, *Lachnospiraceae*, *Bifidobacterium*, *Akkermansia*, *Porphyromonadaceae*, *Desulfovibrio*, *Parabacteroides*, and *Turicibacter*, which were also the dominant taxa in the mice at baseline (Fig. 5C–D), suggesting partial restoration of the gut microbiome profile in mice that survived the *C. difficile* infection. The bacterial taxonomic profile of the mice that were euthanized due to reaching the humane endpoint was consistently dominated by *Escherichia/Shigella*, *Enterococcus*, *Clostridioides*, *Bacteroides*, *Parasutterella*, and *Parabacteroides* (Fig. 5C–D). Except for the genus *Clostridioides*, these taxa were also among the more abundant before *C. difficile* infection, which indicated that the treatments at this time point had not restored the gut microbiome sufficiently after the antibiotic treatment. Two mice treated with FVT-PyT were colonized with 5–30% relative abundance of *Salmonella *(Fig. S7), which may have contributed to increased disease severity leading to euthanasia of these two mice. The potential bacterial engraftment from the FMT inoculum to the FMT treated mice was analyzed at 16S rRNA gene amplicon level. The FMT treated mice that survived the infection were associated with a relative abundance of approx. 65% that was also found in the FMT inoculum, compared with a relative abundance of approx. 15% in the euthanized mice (Fig. S8A). This potential bacterial engraftment from the FMT inoculum was among others represented by *Clostridium sensu stricto* (Fig. 5C–D).

**Fig. 6:**
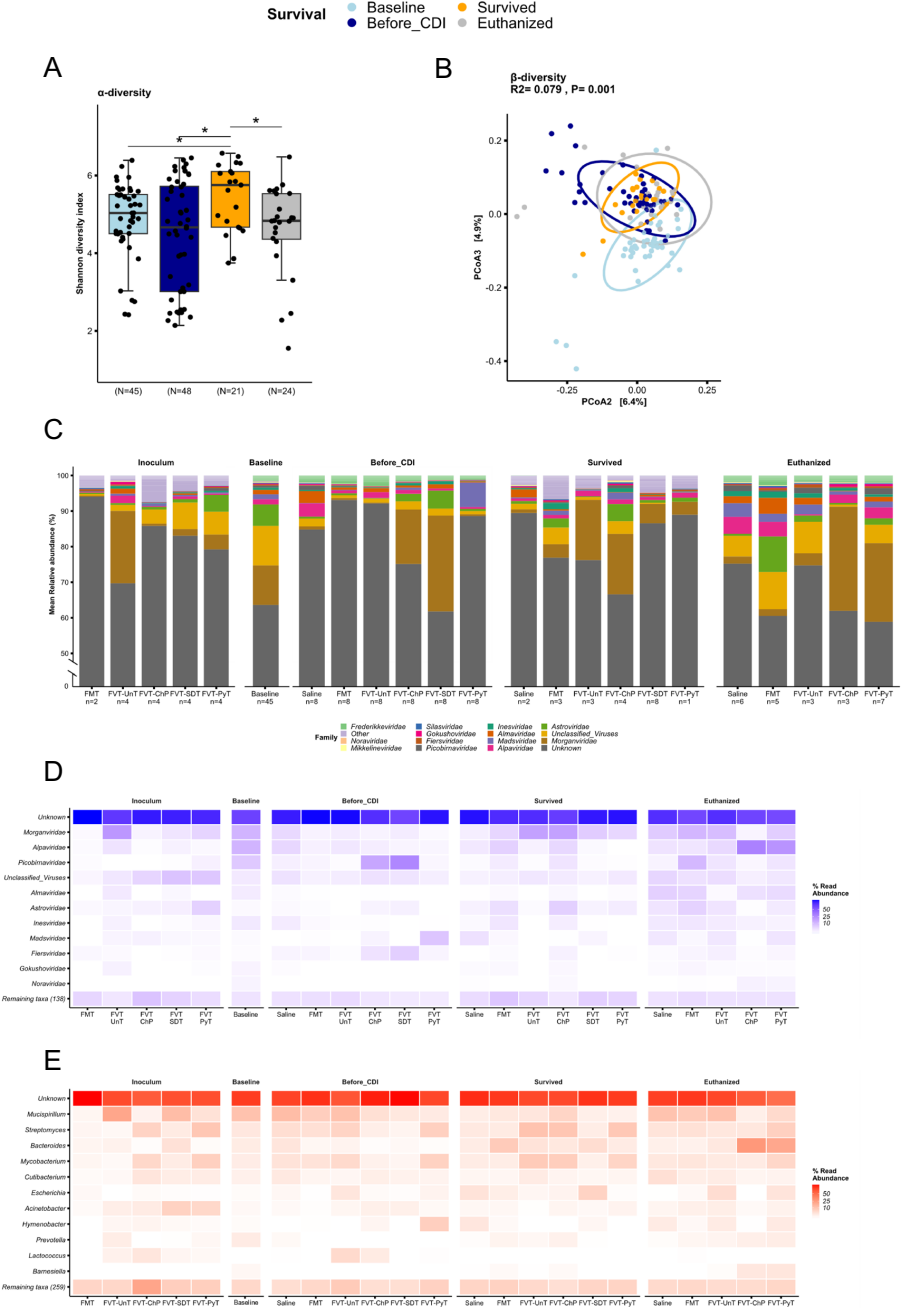
Metavirome analysis based on whole-genome sequencing. **A** The viral Shannon diversity index (α-diversity) and **B** Bray-Curtis dissimilarity based PCoA plot (β-diversity) at baseline (before antibiotic treatment), before *C. difficile* infection (after antibiotic treatment), for the mice that survived *C. difficile* infection (regardless of the treatment), and the euthanized mice. **C** Bar plot showing the relative abundance in percentage of the viral taxonomy (normalized on the basis of transcripts per million, TPM). **D** and **E** heatmaps illustrating the relative abundance in percentage of viruses and the bacterial hosts that are predicted on the basis of the viral sequences, respectively. The “n” below the bar plots highlights the number of mice of which the taxonomical average was based on. *p < 0.055

The viral composition and diversity of the mice that survived the infection were significantly different (*p* < 0.05) compared with the baseline, before *C. difficile* infection (Fig. 6A–B). The dominant viral taxa in all groups at all time points represented *Morgan-*, *Astro-*, *Alpa-*, *Mads-*, *Alma-*, and *Inesviridae*, while more than 60% of the viral relative abundance could not be taxonomically assigned (Fig. 6C–E). The viral engraftment from the FVTs was also investigated at the viral metagenome level (viral contigs). Except for the FVT-UnT, the FMT/FVT treated mice that survived the infection were engrafted with a higher relative abundance of viral contigs that were also found in the different FMT/FVT inoculums, compared with the euthanized mice (Fig. S8B). This observation further indicated that the transfer of phages is likely associated with the treatment efficacy.

**Fig. 7:**
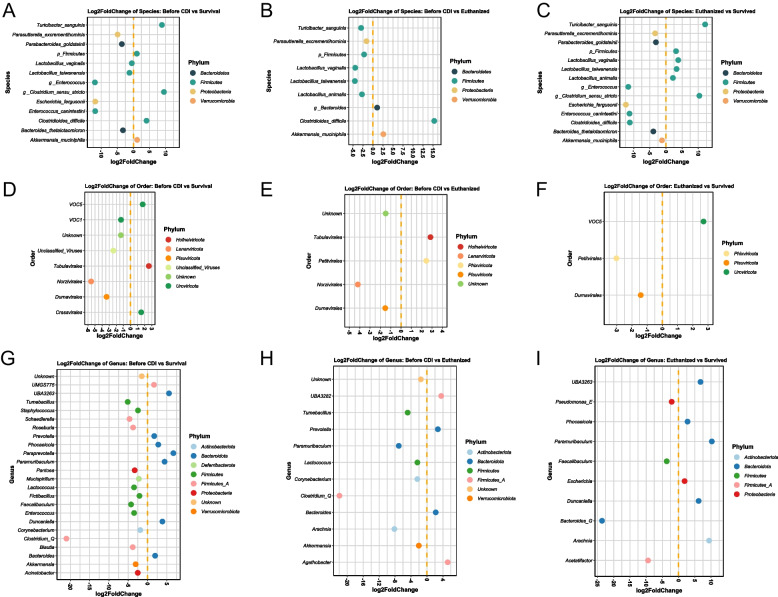
Differential abundance of significantly (*p* < 0.05) different **A**–**C** bacterial taxa, **D**–**F** viral taxa, and **G**–**I** predicted bacterial hosts based on the viral sequences showing three comparisons: microbial taxa before *C. difficile* infection versus survived or euthanized mice and the euthanized versus survived mice

Differential abundance testing was used to characterize the most significant gut microbiome changes of both the bacterial and viral component (relative abundance > 1% and *p* < 0.05) from the time before *C. difficile* infection (after antibiotic treatment) until the mice were euthanized or survived until the study termination (Fig. 7A–C). The mice that survived had significantly increased (*p* < 0.05) their relative abundance of bacterial taxa belonging to *Turicibacter*, *Clostridium sensu stricto*, *Akkermansia*, and *Clostridioides* and a decrease in *Parasutterella, Parabacteroides, Enterococcus, Escherichia,* and *Bacteroides thetaiotaomicron* relative to before they were infected with *C. difficile* (Fig. 7A). The increase in *Clostridioides* is likely due to the persistence of *C. difficile* in the mice which also was detected by the quantitative analysis of *C. difficile* (Fig. 4C). The euthanized mice had significantly increased (*p <* 0.05) their relative abundance of especially *Clostridioides* (15 log_2_ fold change), *Akkermansia*, and *Bacteroides* and a decrease in *Turicibacter*, lactobacilli, and *Parasutterella*, relative to before they were infected with *C. difficile* (Fig. 7B).

With regard to the phages (Fig. 7D–F), the surviving mice were characterized by an increase in viruses (*Vincent*-, *Sonia*-, *Rigmor*-, *Morgan*-, *Freja*-, *Ella*-, and *Christianviridae*) belonging to the viral order of *Crassvirales* and *Tubulavirales* relative to before they were infected with *C. difficile* (Fig. 7D), whereas the euthanized mice mainly had increased their relative abundance of phages (*Nora*-, *Ines*-, *Gokusho*-, and *Alpaviridae*) belonging to the order *Petitvirales* (Fig. 7E). The viral metagenomes were used to predict potential bacterial hosts (Fig. 7G–I) with a recently developed machine learning framework that utilizes six different host prediction approaches [67]. Compared with the time point before *C. difficile* infection, the mice that survived the infection were characterized by a significant increase in (*p* < 0.05) of phages predicted to infect *Prevotella*, *Phocaeicola*, *Paraprevotella*, *Paramuribaculum*, *Duncaniella*, and *Bacteroides* members and a decrease in vira predicted to infect *Tumebacillus*, *Staphylococcus*, *Schaedlerella*, *Roseburia*, *Lactococcus*, *Fictibacillus*, *Faecalibacterium*, *Enterococcus*, *Clostridium*, *Blautia*, *Mucispirillum*, *Pantoea*, *Acinetobacter*, and *Akkermansia* (Fig. 7G). In contrast, the mice that were euthanized showed only an increase in phages predicted to infect *Prevotella*, *Bacteroides*, and *Agathobacter* and a decrease in *Tumebacillus*, *Lactococcus*, *Clostridium*, *Paramuribaculum, Corynebacterium*, *Arachnia*, and *Akkermansia* (Fig. 7H). The overall host-phage relations between the relative abundance (> 0.1%, *p* < 0.05) of bacterial zOTUs and vOTUs (viral contigs) were assessed using Spearman’s correlations which showed clear clustering patterns (Fig. S9).

A bacterial and viral cluster A representing the taxa of *Enterococcus*, *Salmonella*, *Clostridioides difficile*, *Escherichia fergusonii*, *Clostridium cocleatum*, *Bacteroides thetaiotaomicron*, and *Parasutterella* positively correlated with mainly unknown viruses. Another bacterial and viral cluster B representing the taxa of *Prevotella*, lactobacilli, *Turicibacter*, *Clostridium*, *Porphyromonadaceae*, *Lachnospiraceae*, *Bacteroides*, *Bifidobacterium*, and *Coriobacteriaceae* were positive correlated also with mainly unknown viruses. The bacterial genera in cluster B were associated with the mice that survived the infection; hence, the unknown phages in this cluster may represent phages that had positively impacted the restoration of the gut microbiome compared with baseline (Fig. S9). However, due to the limited viral classification, it was not possible to detect clear evidence of specific host-phage relations that were driving the observed curative effects of FVT-UnT, FVT-SDT, and FVT-ChP.

Based on the above, it could be hypothesized that phages transferred along with the FVT-SDT treatment may have contributed to increased gut microbiome resilience against the *C. difficile* infection after the antibiotic treatment and thereby impacting the mice’s ability to fight off the *C. difficile* infection. However, it remains uncertain whether the gut microbiome profile of the surviving mice had been similar in gut microbiome composition to that of the euthanized mice at a certain point of the *C. difficile* infection.

The eukaryotic viral profile was similar among the different FVT inoculates and constituted 0.1–3.0% of the total relative abundance and mainly represented RNA viruses (Fig. S10). However, it is important to note that the taxonomic resolution of eukaryotic viruses was insufficient to differentiate between treatments or outcomes in relation to the relative abundance of eukaryotic viruses (Fig. S10). It should also be emphasized that inactivation by dissolving the viral envelope or inhibiting replication of viruses in the FVT inoculums does not exclude the detection of viruses through sequencing, and the metavirome analysis of FVT-SDT and FVT-PyT can therefore not be used for validation whether specific viruses are inactivated or not.

## Discussion

Here, we have developed methodologies to address the challenges of donor variability and risk of transferring pathogenic microorganisms when using FMT or FVT for treating gut-related diseases. A *C. difficile* infection mouse model was used as proof-of-concept. Inactivation of enveloped viruses through solvent/detergent treatment emerged as the superior method to modify fecal viromes while preserving treatment efficacy against *C. difficile* infection. The systemic inflammatory response observed during *C. difficile* infection is driven by *C. difficile* toxins that increase gut tissue permeability [74, 75], making the gut more susceptible to other microbial infections [76]. Therefore, transferring untreated fecal donor viromes (including intact eukaryotic viruses) may result in additional inflammation due to microbes translocating through the damaged intestinal tissue. The majority of eukaryotic viruses are enveloped [28, 29]. Hence, considering the promising prevention efficacy of *C. difficile* infection in the FVT-SDT group, treating fecal viromes with solvent/detergent prior to transfer to patients may be particularly relevant for diseases that are characterized by increased gut tissue permeability [76].

The lowest survival rate was observed with the RNA targeting compound pyronin-Y. During the initial evaluation of pyronin-Y’s ability to inactivate RNA phages, it became evident that DNA phages were also affected. The pyronin-Y treatment may have caused a reduction in phage activity, which could have impacted the efficacy of the treatment. This would align with several studies emphasizing the importance of high phage titers for successful treatment outcomes [77–80], and phages may play an important role in restoring gut microbiome balance following FMT or FVT [13, 15–17, 19, 26, 44, 81].

FMT is linked to a treatment efficacy above 90% in preclinical [32] and clinical *C. difficile* infection studies [5]; however, the survival rate of FMT treated mice was unexpectedly observed as similar to the saline group. The structure of the animal model does not allow us to assess whether this observation has biological relevance or represents a by-chance finding. Instead, we speculate in two potential explanations. First, the FMT inoculum contained approx. 20% of *Clostridium sensu stricto*, which has been associated with *C. difficile *positive calves [82] and to diarrhea in pigs [83]. The relatively high abundance of *Clostridium sensu stricto* in the FMT inoculum may have counteracted the curative effects typically associated with FMT [13, 32]. While the FMT and FVT inocula originated from the same donor material, the removal of bacteria during FVT processing may explain the higher survival rates observed with FVT-UnT, FVT-ChP, and FVT-SDT compared to FMT. This also suggests that even unsuitable fecal donor material for FMT could potentially be suitable for FVT; thus, FVT-based treatments may have the potential to increase the probability in finding eligible donors, which have been reported as a challenging element of clinical FMT studies [7]. Secondly, a particularly controversial speculation could be that our FMT suspension was handled, sampled, and prepared anoxically inside an anaerobic chamber and stored/suspended in anoxic glycerol/PBS solutions. This is in strong contrast to the preparation of traditional FMT [84], which typically exposes the donor material to oxygen throughout the various preparation steps, from sampling to the final FMT product. It is well-acknowledged that the bacterial gut microbiome component mainly consists of strict and facultative anaerobes [85]. Therefore, it could be speculated that the regular oxic handling of donor material used for FMT unintentionally kills a vast number of oxygen-sensitive bacteria, reducing the load and diversity of viable bacterial cells. This, in turn, may decrease the chance of additional infections or other microbes translocating through damaged intestinal tissue that potentially could cause additional inflammation and tissue damage. In contrast, it would be expected that our anoxically handled FMT had a higher load and diversity of viable strict anaerobic bacteria [86, 87], which may have counteracted the effect of the transferred enteric phages. However, it requires further studies to either confirm or reject this speculation.

Mice treated with FVT-UnT, FVT-ChP, and FVT-SDT showed a significant decrease in *C. difficile* abundance compared to those treated with saline, FMT, and FVT-PyT. We believe that phages transferred along with the FMT/FVT play a role in allowing commensal bacteria associated with a healthy state to compete with the infectious *C. difficile* strain, as well as commensal bacteria that can act as opportunistic pathogens. This was supported by phage engraftment from the FMT/FVT that was associated with mice surviving the infection and a cluster of unknown viruses that were positively correlated with bacteria reflecting a restored gut microbiome. However, the precise mechanisms underlying the gut microbiome modulating effects of FVT remain poorly understood. Nonetheless, several studies have established that the phage donor profile to some extent can be transmissible to the gut of patients suffering from *C. difficile* infections through FMT [13, 44, 81]. Our prior work demonstrated that FVT from lean mice could induce a shift in the gut microbiome composition of obese mice, resembling that of lean individuals [16]. Additionally, we recently illustrated how FVT originating from donors with a relatively high abundance of *A. muciniphila* could significantly elevate the relative abundance of the endogenous *A. muciniphila* in recipient mice [21], and another study showed that autochthonously transferred FVT is protecting against stress-associated behavior in mice [33]. These observations from independent research groups imply that the phenotypic traits of FVT donors may be transferred to recipients, possibly driven by the inclination of phages to establish ecosystems similar to their origins. This process may involve cascading events [26], as illustrated in a gnotobiotic mouse model where phage infections indirectly influenced the bacterial balance [88]. Consequently, the more complex viral community of FVT could similarly impact the bacterial ecosystem that influences the metabolome resulting in systemic changes, as seen in our previous study [16]. While the notion of such effects may seem counterintuitive given the commonly held belief in the strain-specific nature of phages, a recent study proposed that phages could interact with distantly related microbial hosts [89]. Phage satellites have also been suggested to contribute to broader host ranges [90, 91]. Furthermore, the transfer of potentially beneficial metabolic genes from temperate phages to their bacterial hosts may enhance host competitiveness and contribute to overall microbiota changes [92, 93]. These findings align with recent research demonstrating the significant influence of nutritional and host environments on the community ecology [94], suggesting that cascading events initiated by FVT could catalyze changes in the host environment. Beyond the bacteria-phage relations, the role of the immune system in gut health should not be underestimated. Recent evidence suggests that phages interact with the immune system through mechanisms like TLR3 and TLR9 [95, 96] or other mechanisms resulting in the uptake by mammalian cells [97, 98]. A recent review has summarized the current understanding of phage immunogenicity, highlighting parallels with eukaryotic viruses [99, 100]. Thus, stimulation of the immune system may represent another mechanism behind the effects of FVT.

While there were good indications that phages were a key component in the observed effects associated with the FVT-UnT, FVT-SDT, and FVT-ChP treated mice, it remains possible that metabolites or entities with a molecular size above 30 kDa (size cutoff of applied ultrafilter) may have contributed to the observed effects. These molecules could for instance be metabolites from lactobacilli [101], and *Akkermansia* spp. (pasteurized cell cultures) [102], bacteriocins with antimicrobial properties affecting the gut microbiome composition [103, 104], or extracellular vehicles which have been shown to affect immune regulation during pregnancy [105, 106], and may be involved in the etiology of inflammatory bowel diseases [107]. However, considering that most metabolites have a size less than 30 kDa [46, 47], long-term colonization of donor phages in FMT studies [81, 108, 109], phages being associated to the treatment outcome of recurrent *C. difficile* infection [44, 81], no reported effects of heat-treated FVT controls [15, 110], and studies reporting beneficial effects of FVT in different etiology regimes [12, 13, 15, 16, 19], it suggests that the viral component of FVT constitutes an important role. In addition, it could be speculated that the solvent-detergent treatment (FVT-SDT) likely has dissolved a certain fraction of lipid based extracellular vesicles and thereby further diminished their potential role in the treatment outcome using FVT-SDT.

The FVT preparation protocol does not remove microorganisms or other entities that can pass through the applied 0.45 µm filtration. Bacterial endospores exhibit a size range, with some as small as 0.25 µm, although their typical dimensions surpass 0.8 µm [111, 112]. Similarly, certain bacterial species within the taxa of *Mycoplasma*, *Pelagibacter*, and *Actinobacteria* can attain sizes as small as 0.2 µm [113–118], while most bacteria generally range in length from 1 to 10 µm [119]. Hence, the use of a 0.45 µm sterile filtration is anticipated to eliminate the vast majority of bacterial cells and spores. This was supported by both the notably low colony forming unit (CFU) counts observed in the FVTs under the investigated conditions (Table S2) and the 16S rRNA gene profile of the FVTs showing low read counts and/or no gut-associated bacteria in 3 out of 4 FVTs (Fig. S11). Choosing a smaller pore size like 0.22 µm can minimize contamination of bacteria but will cause in exclusion of large viruses and phages [120] and have been shown to negatively affect the abundance of common phages [121], thus making 0.22 µm filtration an undesirable solution for FVT preparation. The abundance and significance of nano sized bacteria in gut health remain sparsely investigated [122]. We can therefore neither confirm nor definitively rule out that these bacteria potentially have influenced the treatment outcomes of the FVTs.

The application of multiple displacement amplification (MDA) for 1.5–2.0 h has been reported to compromise quantitative analysis of metagenomes by overestimating the abundance of ssDNA sequences [123, 124]; however, it has recently been shown that decreasing the time of whole genome amplification to 30 min accommodates this bias to a level where it remains valid to compare inter-sample relative abundance of viruses [61]. The taxonomical classification of eukaryotic viruses mainly detected RNA viruses, while only one DNA virus was detected. This would be in accordance with eukaryotic viruses being dominated by RNA viruses [28, 29]. Phages are generally species or strain specific [14], but the limited bacterial taxonomical resolution, which is associated with 16S rRNA gene amplicon sequencing, restricts predicted host-phage correlations to the genus level of the bacteria.

A recent study showed that *C. difficile* senses the mucus layer since it moves towards the mucin glycan components due to chemotaxis, and that mucin-degrading bacteria like *Akkermansia muciniphila*, *Bacteroides thetaiotaomicron*, and *Ruminococcus torques* allow *C. difficile* to grow when co-cultured in culture media containing purified MUC2 but without glucose, despite *C. difficile* lacks the glycosyl hydrolases needed for degrading mucin glycans [125]. Interestingly, coexistence of these bacterial taxa may explain why the euthanized mice tended to lose most of the commensal bacteria like *Prevotella*, lactobacilli, *Turicibacter*, and *Bifidobacterium*, while *Akkermansia* and *Bacteroidetes* persisted. Preventive use of frequent administration of high doses of *A. muciniphila* has been shown to alleviate *C. difficile* infection associated symptoms in a similar mouse model [126], which together points in the direction of a coexistence rather than a symbiosis between *C. difficile* and mucin degrading bacteria.

The high mortality rate associated with the included *C. difficile* VPI 10463 strain makes it valuable for assessing the main endpoint parameter of survival probability related to various FVTs. Euthanizing animals that reached the humane endpoint at different time points had naturally an impact on the statistical power and introduced challenges in evaluating time dependent parameters such as cytokine profiles, histopathology, and comparable time series analysis of gut microbiome recovery. On the contrary, it is possible that if the surviving mice exhibited comparable histology, *C. difficile* abundance, cytokine profiles, toxin levels, and gut microbiome profiles, they would have reached the humane endpoint at a similar time point as the euthanized mice. The animal model was designed as such to adhere to the 3Rs principles [39] (replacement, reduction, and refinement) by limiting the number of mice per group to eight instead of employing several termination points for all treatment groups. In addition, the group size was evaluated as sufficient for screening the survival probability associated with the different FMT/FVT treatments. It would have provided additional insights of the role of phages in the FVT treatment of the *C. difficile* infection if UV and/or heat-treated FVT controls were included in the design of the animal model. However, due to the severity of the *C. difficile* infection model applied, it would not accommodate the principle of 3Rs (reduce) [39] to include additional animals considering that two previous studies have shown no effect of heat-treated FVT controls [15, 110]. Thus, it would be extremely relevant to include such controls in future studies using animal models causing less severity in disease development.

As an alternative to phage-based therapies to restore a dysbiotic gut microbiome, a recent bacterial consortium of *Firmicutes *spores (SER-109) has been approved by the FDA to treat rCDI [127, 128], which highlights the potential of also using bacterial consortia in modulation of the gut microbiome. The treatment efficacy of SER-109 was found to be 28% percentage points (88%) increased compared with placebo (60%) [127], while regular FMT in another study showed 57% percentage points (90%) enhanced treatment efficacy compared with placebo (33%) [5]. This suggested that FMT may still be the preferred treatment strategy for rCDI depending on the patient group. Furthermore, the role on treatment outcome of induced prophages originating from the bacteria in the SER-109 consortium remains to be addressed. Additional studies are therefore necessary to be conducted for comparing phage-based treatments with FMT and bacterial consortia like SER-109.

A major challenge of *C. difficile* infection is the risk of recurrent infections [6]. It is therefore interesting to note that seven out of eight FVT-SDT treated mice showed non-detectable of *C. difficile* at termination, indicating a decrease in the risk of recurrent infections when treated with a solvent/detergent modified fecal virome. The inherent challenges of variability and reproducibility in fecal donor material exist for both FMT and FVT [7, 8]. Two independent studies have demonstrated how propagation of fecal inoculum in a chemostat fermentation holds the potential for reproducing the enteric viral component [54, 129]. Interestingly, the treatment efficacy and decrease in *C. difficile* infection associated symptoms were also pronounced in mice treated with the chemostat-propagated enteric virome. Therefore, it could be argued that the solvent/detergent methodology of fecal viromes, already approved by WHO as a safe procedure for treating blood plasma [50], holds the potential to complement FMT in the treatment of *C. difficile* infection in the short-term perspective. In the long-term perspective, a cost-effective, standardized, and reproducible chemostat-propagated enteric phageome for *C. difficile* infection treatment may also have tremendous potential for phage-mediated treatment of other diseases associated with gut dysbiosis.

## Conclusion

The hypothesis of this proof-of-concept study was that different modifications of FVT had the potential to address the challenges of donor variability and infections risks that are associated with FVT/FMT. Especially, two FVT modification strategies showed an effect in limiting the colonization of *C. difficile* in the infected mice and thereby increased their chance of survival. Inactivation of enveloped viruses through solvent/detergent treatment of the FVT appeared as a superior method to address the infection risks while preserving treatment efficacy against *C. difficile* infection. Also, the chemostat-propagated FVT showed promising potential as a methodology to address both donor variability and the infection risks, thus overall confirming our initial hypothesis. Due to the natural limitations associated with the simplicity of the study, these results encourage additional preclinical studies to further validify the translatability and relevance of applying these concepts of FVT treatments in clinical settings.

### Supplementary Information

**Additional file 1: Fig. S1.** Mouse body weight measured at arrival (day 0), just before antibiotic treatment (day 8), just before *C. difficile* infection (day 15), just before 1^st^ treatment (day 16), just before 2^nd^ treatment (day 18), one week after 2^nd^ treatment (day 23), two weeks after 2^nd^ treatment (day 30), and termination (day 35). **Fig. S2.** A) Bacterial calibration curve showing the correlation between OD_600nm_ and colony forming unite per mL (CFU/mL) of *C. difficile* VPI 10463 and B) and C) phase contrast microscopy confirming the expected cell morphology of *C. difficile* VPI 10463. D) CFU counts in technical replicates of the applied *C. difficile* inoculum and the total CFU that was transferred to each mouse. E) Epifluorescence microscopy images of the different applied FVT viromes, before being normalized to similar virus-like particles(VLP)/mL concentrations that were stained with SYBR Gold to count the VLP/mL. Images were taken using a 100x magnification objective. Scale bar for the bacteria is 4 µm in C) and D) and for VLPs in E) the scale bar is 1 µm. **Fig. S3.** Flow diagram illustrating the origin of the intestinal donor content and the processing steps to generate the FVTs with different methodologies. Two C57BL/6NRj mice were euthanized due to malocclusion-associated malnutrition. Then 52 mice from 3 different vendors were sacrificed and their intestinal content from cecum and colon was collected, however, 34 mice were sampled in oxic conditions, while 18 mice were sampled in anoxic conditions to maintain the viability of the strict anaerobic bacterial gut microbiome members. The atmospheric conditions were maintained throughout the process. Regardless of vendor, the intestinal content was mixed. Virome separation was performed for the oxic handled fecal mixture and the extracted fecal viromes were divided in three aliquots that represented 1) the untreated fecal virome (FVT-UnT), and 2) the further processing with solvent/detergent treatment (FVT-SDT) or 3) pyronin-Y treatment (FVT-PyT). Anoxic handled fecal mixture was divided in two aliquots for use as fecal microbiota transplantation (FMT) in the CDI mouse model, or as inoculum for fecal virome propagation in a chemostat setup (FVT-ChP). Also, the FVT-ChP underwent virome separation to remove most metabolites as well as bacteria and other larger microbes. **Fig. S4.** Evaluation of CDI symptoms. A) Representative histology images of cecum tissue sampled at either termination of the study or when mice were euthanized due to reaching the humane endpoint. Asterisks = immune cell infiltrates, arrowheads = congested submucosal blood vessels, arrows = volcano lesions contributing to pseudo-membrane formation. Histology image scale bar = 300 µm. B-F) Graphs showing the qualitative visual assessment of the health status of individual mice during the monitoring. The evaluations were based on the level of physical activity (i.e. decreased spontaneous or provoked activity), level of watery feces, body posture (hunching), and whether their fur was kept clean or not. The score of 0 (healthy), 1 (mild symptoms), 2 (clear symptoms), or 3 (mice with score 2 that did not show improvement within next checkup were euthanized). The mouse ID is indicated below each graph. H-J) Showing violin plots of each of the three parameters evaluated in the histopathology assessment of the cecum tissue. Numbers above shows the p-value. **Fig. S5.** Bacteriome (16S rRNA gene amplicon sequencing) and metavirome (whole-genome sequencing) analysis at three time points; Baseline (before antibiotic treatment), before CDI infection (after antibiotic treatment), and at termination (euthanized/survived). The treatment groups represents the average regardless of whether the mice survived the infection until termination or were euthanized before termination. A) & C) The bacterial and viral Shannon diversity-index (α-diversity) and B) & D) Bray-Curtis dissimilarity based PCoA plot (β-diversity). Volcano plots showing the differential relative abundance for E) the bacterial and F) viral taxa compared with the saline control. **Fig. S6.** Bacteriome (16S rRNA gene amplicon sequencing) and metavirome (whole-genome sequencing) analysis at three time points; Baseline (before antibiotic treatment), before *C. difficile* infection (after antibiotic treatment), and at termination of mice that either survived until termination or were euthanized regardless of the treatment. A) The bacterial Shannon diversity-index (α-diversity) and B) Bray-Curtis dissimilarity based PCoA plot (β-diversity). C) Heatmap illustrating the bacterial relative abundance in percentages of the dominating bacterial taxa that was associated to the mice that survived versus euthanized. D) The viral Shannon diversity-index and E) Bray-Curtis dissimilarity based PCoA plot. F) and G) Heatmaps illustrating the relative abundance in percentages of, respectively, the viral taxonomy and bacterial hosts that are predicted based on the viral sequences. **Fig. S7.** Taxonomical bar plot showing the relative abundance in percentages of each individual mouse in the FVT-PyT treated group, both before the *C. difficile* infection (after antibiotic treatment) and when the mice were either euthanized or survived. Mouse No 45 and 47 had a relatively high abundance of *Salmonella* that was not observed in the other mice. **Fig. S8.** The potential bacterial and viral engraftment from the FMT/FVT inoculums to the treated mice were analyzed at A) 16S rRNA gene amplicon and B) viral metagenome level (viral contigs). The gut microbiomes were divided into donor origin and the different timepoints of baseline (before antibiotic treatment), before *C. difficile* infection (after antibiotic treatment), and when the mice survived until termination or were euthanized. There was an overlap of amplicons/viral contigs found in both the FVT inoculum and in the mice at the timepoints before the treatment, hence this analysis only partly describes the bacterial and viral engraftments. **Fig. S9.** Spearman’s correlation analysis of the bacterial and viral relative abundance. Cluster A and B marks bacterial and viral positive correlations. **Fig. S10.** Metavirome analysis of only eukaryotic viruses (that could be classified as such) based on whole-genome sequencing at three time points; baseline (before antibiotic treatment), before *C. difficile* infection (after antibiotic treatment), and at termination of mice that either survived until termination or were euthanized. A) The viral Shannon diversity index (α-diversity) and B) Bray-Curtis dissimilarity based PCoA plot (β-diversity). C) Box plot showing the relative abundance in percentages of eukaryotic viruses in the different FMT/FVT inoculums. D) Heatmap illustrating the relative abundance of eukaryotic viral taxonomy. E) Box plot showing the relative abundance in percentages of eukaryotic viruses in the differently treated mice over time. The applied FMT and different FVTs inoculums were spiked with a defined phage mock community as a technical control of the applied protocols to detect ss/dsRNA and ss/dsDNA viruses. **Fig. S11.** 16S rRNA gene amplicon sequencing of the FMT/FVT inoculums to assess their bacterial profile. Bar plot showing A) the number of reads detected and B) the number of observed bacterial species (α-diversity), and C) the relative abundance in percentage of the bacterial taxa detected.

**Additional file 2: Table S1.** List of included bacterial and phage strains along with relevant strain-specific information and growth conditions that was used for plaque assays. The same phages were also used as a mock community (positive control) and spiking for the metavirome sequencing where the phages where normalized to 10^6^ PFU/mL for each phage. **Table S2.** The FVTs used in the current study were anaerobically incubated on non-selective Gifu anaerobic medium (GAM) for 14 days at 37 °C. Here we observed the below colony forming units (CFU) counts for each of the applied FVTs. **Table S3.** List of components and their associated concentration (g/L) in the growth medium used for chemostat propagation of the chemostat propagated fecal virome (FVT-ChP). **Table S4.** List of antibiotic (AB) water consumption. The volumes were calculated as the average of each cage.

**Additional file 3.** The ARRIVE Essential 10 checklist.

## Data Availability

All data associated with this study are present in the paper or the supplementary materials. All sequencing datasets are available in the ENA database under accession number PRJEB58777.
